# Inflammation, Immune Senescence, and Dysregulated Immune Regulation in the Elderly

**DOI:** 10.3389/fragi.2022.840827

**Published:** 2022-04-27

**Authors:** Carey Shive, Pushpa Pandiyan

**Affiliations:** ^1^ Louis Stokes Cleveland VA Medical Center, United States Department of Veterans Affairs, Cleveland, OH, United States; ^2^ Case Western Reserve University, Cleveland, OH, United States

**Keywords:** inflammaging, elderly, immune senescence, dysregulated tregs, t cell senescence, inflammation

## Abstract

An optimal immune response requires the appropriate interaction between the innate and the adaptive arms of the immune system as well as a proper balance of activation and regulation. After decades of life, the aging immune system is continuously exposed to immune stressors and inflammatory assaults that lead to immune senescence. In this review, we will discuss inflammaging in the elderly, specifically concentrating on IL-6 and IL-1b in the context of T lymphocytes, and how inflammation is related to mortality and morbidities, specifically cardiovascular disease and cancer. Although a number of studies suggests that the anti-inflammatory cytokine TGF-b is elevated in the elderly, heightened inflammation persists. Thus, the regulation of the immune response and the ability to return the immune system to homeostasis is also important. Therefore, we will discuss cellular alterations in aging, concentrating on senescent T cells and CD4+ CD25+ FOXP3+ regulatory T cells (Tregs) in aging

## Introduction

By 2050 it is predicted that 22% of the US population will be > 65 years old and one in six people globally will be over the age of 65 (United Nations DoEaSA, 2019). The decline in immunity with age contributes to poor response to vaccines, reduced recovery from new pathogens, increased cancer rates, and a host of other morbidities. The average global lifespan has increased from 47 to 73 years over the last 70 years (Garmany et al., 2021). Unfortunately, healthspan, the period of life free from chronic disease and disabilities of aging, has not kept pace (Garmany et al., 2021). A summary of the “Collaboration for Non-communicable Diseases” describes four common conditions that account for 80% of global chronic disease-related deaths: cardiovascular disease (CVD), cancer, diabetes, and chronic respiratory disease (Frieden et al., 2020). In the US, the leading causes of death in persons >65 years old from 1999 to 2019 were CVD, cancer, chronic lower respiratory diseases, cerebrovascular diseases, Alzheimer disease, and diabetes (Centers for Disease Contr, 2020). Elderly patients also suffer from rheumatoid arthritis, sarcopenia and frailty (Maggio et al., 2006).

Although the German scientist Hanns Kaiser published a number of articles in the 1970s relating inflammation to diseases in the elderly (Kaiser, 1971), the last 20 years have seen a burst in the study of the aging immune system, inflammation and the associated diseases. One of the most iconic studies in aging and immunity was the OCTO and NONA longitudinal study of healthy 80–90 year old people that took place in Jonkoping Sweden in the 1990s (Wikby AJ and Ferguson, 2003). These studies were unique for several reasons. First, they were longitudinal studies of elderly individuals. Second, the 80- and 90-year-olds were very healthy. As people age, they naturally acquire more disease and it becomes increasingly difficult to distinguish the effects of disease vs. aging on the immune parameters being measured. To overcome these confounders, the OCTO and NONA immune longitudinal study was a community population-based study that continually and carefully evaluated individual health parameters. Study participants had normal cognition, were not on drugs that would influence their immune responses, and were non-institutionalized (Wikby AJ and Ferguson, 2003). Several important findings resulted from these studies; 1) the establishment of an Immune Risk Profile (IRP) based on altered CD4/CD8 T cell ratios (decreasing CD4^+^ T cell numbers and often increasing CD8^+^ T cell numbers) (Wikby et al., 1998) and 2) germane to this review, the discovery of a link between elevated plasma IL-6 levels, mortality and IRP in the very old (Wikby et al., 2006).

An optimal immune response requires the appropriate interaction between the innate and the adaptive arms of the immune system as well as a proper balance of activation and regulation. After decades of life, the aging immune system is continuously exposed to immune stressors and inflammatory assaults that lead to immune senescence (Salama et al., 2014; Aiello et al., 2019; Di Micco et al., 2021). In this review, we will discuss inflammaging in the elderly, specifically concentrating on IL-6 and IL-1β in the context of T lymphocytes, and how inflammation is related to mortality and morbidities, specifically cardiovascular disease and cancer. Although a number of studies suggests that the anti-inflammatory cytokine TGF-β is elevated in the elderly, heightened inflammation persists. Thus, the regulation of the immune response and the ability to return the immune system to homeostasis is also important. Therefore, we will discuss cellular alterations in aging, concentrating on senescent T cells and CD4^+^ CD25 ^+^ FOXP3+ regulatory T cells (T_regs_) in aging.

## Cytokines in Aging

### What Is Inflammaging?

Inflammaging is a phenomenon of inflammatory pathogenesis characterized by chronic low-grade inflammation, and is a significant risk factor for morbidity and mortality in elderly people. Claudio Franceschi first coined the term “inflammaging” in the manuscript “*Inflamm-aging*.

*An Evolutionary Perspective on Immunosenescence*” (Franceschi et al., 2000). Although written from an evolutionary perspective, the authors recognize the continuous response to “stressors” in the aging immune system and the fundamental importance of the innate immune system and subclinical, elevated chronic inflammatory mediators (cytokines, soluble receptors, chemokines, coagulation factors, stress hormones) (Franceschi et al., 2000). They also recognize the increasing presence of circulating IL-6 and its association with mortality in the elderly. However, they also stress that elevated plasma IL-6 levels are present in long-living centenarians as well (Franceschi et al., 2000).

### Innate Cytokines

Innate immunity and the acute phase response of the immune system are altered with advancing age. The immediate response to infection and/or tissue damage involves liver production of acute phase proteins like C-reactive protein (CRP) and serum amyloid A (SAA); fever, often triggered by the pyrogenic cytokine IL-1β; and granulocytosis (Bruunsgaard et al., 2001). Cytokines associated with innate immunity and the acute-phase response (IL-6, IL-1β, TNFα) can be produced by innate cells of the immune system, monocytes, macrophages, dendritic cells, and also by tissue-specific cells such as endothelial and epithelial cells, adipose cells, and fibroblasts (Freeman et al., 2016a). Chronic expression of IL-6, IL-1β, and TNFα is associated with pathology and mortality in the elderly (Bruunsgaard et al., 2001). Acute-phase serum proteins such as fibrinogen, albumin, and ceruloplasmin are all related to cardiovascular disease risk (Kritchevsky et al., 2005). Aged T cells capable of responding to these cytokines and proteins and orchestrating chronic inflammatory responses also have pathogenic potential in cardiovascular diseases (Yu et al., 2016).

#### Interleukin-6

The cytokine most often associated with aging and mortality is IL-6. In fact, it has been dubbed “a cytokine for gerontologists” (Ershler, 1993). Furthermore, elevated plasma levels of IL-6 (and to a lesser degree, TNFα) predicted mortality in studies of relatively healthy >80 year olds (Bruunsgaard et al., 2001; Wikby et al., 2006). IL-6 is extremely pleiotropic, not only is it produced by numerous immune and tissue cell types (T cells, B cells, monocytes, macrophages, dendritic cells, endothelial and epithelial cells, keratinocytes, fibroblasts, astrocytes, microglia, adipose cells, and muscle cells (including cardiomyocytes)), it has both inflammatory and anti-inflammatory functions. During the acute phase response to injury or infection, TNFα and IL-1β are produced and they, in turn, induce the production of IL-6. IL-6 then stimulates the liver to produce acute-phase proteins like CRP and SAA. When IL-6 binds to the IL-6 receptor and its co-receptor, gp-130, on the surface of a cell, this classic signaling is usually anti-inflammatory and can upregulate suppressor of cytokine signaling molecule 3 (SOCS3) which results in feedback inhibition (Ershler and Keller, 2000; Maggio et al., 2006; Greenhill et al., 2011). IL-6 also controls the pro-inflammatory signaling of IL-1β and TNFα by inducing the production of IL-1 receptor antagonists (IL-1Ra) (Ershler and Keller, 2000; Steensberg et al., 2003; Rea et al., 2018) and sTNF receptors, respectively (Tilg et al., 1994; Rea et al., 2018). Lastly, IL-6 is involved in wound healing and (in this capacity) contributes to fibrosis, possibly by upregulating the TGF-β receptor (Luckett-Chastain and Gallucci, 2009) and augmenting TGFβ signaling (Zhang et al., 2005). IL-6 can also bind to its soluble receptor and then bind to coreceptor gp130 that is ubiquitously expressed on many cell types. This “trans-signaling” results in sustained high levels of STAT3 and subsequent inflammation (Maggio et al., 2006; Greenhill et al., 2011).

Because IL-6 has so many pleiotropic effects both pro-inflammatory and anti-inflammatory, it is not surprising that it is associated with morbidity and mortality in aging. An early study of IL-6 in the elderly found that elevated levels of plasma IL-6 with age were associated with the prevalence of heart attack, high blood pressure, cancer, and functional disability (3 indexes of daily living were measured) (Cohen et al., 1997). A study of elderly veterans found that plasma levels of IL-6 and soluble TNF receptors (sTNFR) I and II were higher in frail and pre-fail veterans than non-frail veterans and frailty was more closely associated with inflammation than chronological age (Van Epps et al., 2016). Another large study of 1,293 non-disabled elderly people found that higher levels of circulating IL-6 and CRP were associated with all-cause mortality and specifically, cardiovascular disease (Harris et al., 1999). It is important to recognize that these associations may or may not be predictive, and identifying a causative pathway or mechanism is challenging. Our studies below relating to T regulatory cell dysfunction and T cell senescence suggest possible mechanistic links between IL-6 and morbidities in the elderly.

#### Interleukin 1β

IL-1β and TNFα are the first cytokines released at the sign of damage or disease. Several classic danger-associated molecular patterns (DAMP)s, pathogen-associated molecular patterns (PAMP)s and unique danger signals (lipotoxic fatty acids, ceramides, ATP, ROS, cholesterol and uric acid crystals, K+ efflux, b-amyloid fibrils) are recognized by cells and trigger inflammasome formation, the end result of which is the production of IL-1β and IL-18. The inflammasome is a complex of Nod-like receptors (NLRs), apoptosis-associated speck-like protein containing a CARD (ASC), and pro-caspase-1 that cleaves pro-IL-1β yielding active IL-1β (Dinarello, 2009; Aden and Rosenstiel, 2017).

Circulating levels of IL-1β are notoriously difficult to measure reliably in humans (Koelman et al., 2019). However, in a systematic review and meta-analysis of studies examining plasma levels of IL-1β, IL-6, TNFα, and CRP in elderly patients with depression or Alzheimer’s disease, IL-1β was measured in eleven studies between 2005 and 2018 (Ng et al., 2018). Examination of inflammasome genes, precursors to IL-1β production, may be more reliable than measurement of secreted plasma levels of IL-1β. However, cytokine gene expression does not always parallel cytokine secretion. Yet, Furman et al. found two inflammasome gene modules in older individuals that were persistently elevated and correlated with elevated blood pressure, arterial stiffness, and chronic levels of inflammatory cytokines (Furman et al., 2017). In an elegant study of aged Nlrp3^−/−^ mice, Youm et al. found that the NLRP3 inflammasome is activated in response to age-related DAMPs, but in aged Nlrp3 −/− mice, caspase-1 mediated inflammation is low. Further, they found that the functional decline seen in aged mice was abrogated in older Nlrp3^−/−^ mice (Youm et al., 2013). Their data indicate that NLRP3 inflammasome may promote IL-1β-mediated functional measures of frailty and cognitive decline (Youm et al., 2013).

### Anti-Inflammatory Cytokines

Studies of circulation cytokines in the elderly reveal a paradox. Although the elevation of inflammatory cytokines is associated with inflammaging, elevated circulating anti-inflammatory cytokines are also characteristic in the elderly. A sub-study of the OCTO and NONA longitudinal immune study examined plasma anti-inflammatory cytokine profiles in 138 participants (age 86–94) in comparison to 18 healthy volunteers aged 32–59. They found that IL-6, soluble Intercellular Adhesion Molecule 1 (sICAM-1), and active TGF-β plasma levels were elevated in the elderly (Forsey et al., 2003) compared to plasma levels in the younger (32–59) healthy volunteers. Few other studies have examined plasma TGF-β levels in the elderly (Tominaga and Suzuki, 2019). Although TGF-β is classically considered anti-inflammatory, it is one of the main cytokines contributing to wound healing and fibrosis. TGF-β signaling is known to contribute to cellular senescence and has been associated with aging disorders such as muscle atrophy, obesity, and Alzheimer’s disease (Tominaga and Suzuki, 2019).

Thus, previous studies show that both inflammatory and anti-inflammatory cytokines are elevated in old age. Continuous stressors over a lifetime likely stimulate low-level inflammation (Franceschi et al., 2000; Bruunsgaard et al., 2001; Morrisette-Thomas et al., 2014). In response to this inflammation, anti-inflammatory cytokines are induced in an attempt to return the immune system to homeostasis. A study that used a principal component analysis (PCA) to interrogate soluble immune mediators in the elderly found both inflammatory (IL-6, CRP, TNFα, IL-18) and anti-inflammatory (sTNFRI, sTNFRII, IL-1Ra) mediators that associated with advanced aging (Morrisette-Thomas et al., 2014).

The immune phenotype that results may depend upon the balance of inflammatory and anti-inflammatory cytokines. The study of the “OCTO” cohort that found a link between plasma IL-6 levels and mortality in the very old also identified individuals who moved out of the high IRP group who had elevated plasma levels of both IL-6 and IL-10 (Wikby et al., 2006). Unfortunately, no investigation into the ratio or balance of IL-6 and IL-10 was reported in this study. In a small study of *n* = 29 healthy young adults (<35 years) and *n* = 31 healthy elderly adults (>65 years), plasma levels of TNFα (pro-inflammatory) and IL-10 (anti-inflammatory) were measured at three- time points within 1 year. At each time point, levels of TNFα were higher and IL-10 levels were lower in the elderly (Saurwein-Teissl et al., 2000). A review of cytokines in aging found numerous studies showing elevated innate pro-inflammatory cytokines but the studies examining anti-inflammatory TGF-β levels were contradictory (Minciullo et al., 2016). The authors mention that plasma levels of inflammatory cytokines are elevated even in healthy centenarians, but long-lived people avoid many common age-related diseases, possibly because they maintain a balance between inflammatory and anti-inflammatory responses (Minciullo et al., 2016). Most studies of IL-10 reviewed in this study examined polymorphisms of IL-10. Some studies found that IL-10 correlated with longevity and reduced risk of CVD, however, some showed no correlation (Minciullo et al., 2016).

An alternative explanation for the elevated circulation of both pro-inflammatory and anti-inflammatory cytokines is that the immune regulatory feedback in the elderly is dysfunctional. An *in vivo* human endotoxin challenge model, where young and old patients were given a bolus of LPS, showed acute increases of IL-6, TNFα, and IL-10 in both groups. However, the older patients had prolonged inflammatory activity and a prolonged fever response (Bruunsgaard et al., 2001). *In vivo* studies comparing pneumococcal infections in old and young patients found similar results (Bruunsgaard et al., 2001). This suggests a possible insensitivity to feedback or inhibitory mechanisms or a possible defect in immune regulation. Our studies of T regulatory cells in aged mice and humans suggest that T regulatory cells become dysfunctional in the elderly (see the section below).

Understanding the possible causes of inflammaging will be important when considering possible therapeutics or biologics that may help treat the immune dysfunction that accompanies aging. The ability to intervene early to prevent inflammaging, could prevent the cascade of inflammation and immune dysfunction, thereby attenuating the diseases of aging.

## Aging, Disease, and Inflammation

Treating disease in the elderly is much more complex than treating disease in younger populations because of multiple comorbidities, polypharmacy, and persistent low-grade inflammation, as well as dysregulated immune homeostasis in the elderly. Further complicating the goal to increase healthspan in later years of life is the complex social, mental, economic, and overall care of the elderly population as their “self-care” capacity diminishes (Garmany et al., 2021).

Chronic low-level inflammation underlies all of the diseases mentioned in the introduction. Although some understanding of the inflammatory pathways and mechanisms leading to disease have been described, many studies are primarily associative. Many of the associations between inflammation and disease reflect the reciprocal nature of inflammation, thus a further understanding of the underlying mechanisms is needed to better treat these conditions in the elderly. Here, we will concentrate on age-associated cardiovascular disease and cancer.

### Aging and Cardiovascular Disease

As mentioned in the introduction, in the US the leading cause of death in those over the age of 65 is cardiovascular disease. Atherosclerosis is now recognized as a chronic inflammatory condition (Ross, 1999). An early study found that elevated circulating IL-6 levels in patients >65 years were associated with a history of heart attack (Cohen et al., 1997). Another large study of 1,293 healthy, non-disabled >65-year-old participants, found that plasma levels of IL-6 and CRP were associated with death from CVD (Harris et al., 1999). Levels of plasma IL-6 in this study fell within what has been considered chronic low-level inflammaging (Harris et al., 1999). It is important to recognize that the biomarkers predictive of CVD in middle-aged patients may be different from those found in elderly patients. This was shown in a study that specifically examined biomarkers in patients >65 years old (Kritchevsky et al., 2005). They found that in studies specifically examining elderly participants, plasma levels of IL-6 and TNFα were better predictors of CVD than fibrinogen or CRP levels. Whereas plasma levels of CRP and fibrinogen were good predictors of CVD in middle-aged patients (Kritchevsky et al., 2005). In addition, low-density lipoprotein (LDL) and total serum cholesterol are regularly used as indicators of future cardiac events in middle-aged patients, but in the elderly, they are weak predictors of future cardiac events (Kritchevsky et al., 2005).

A persistent question in CVD “biomarker” research is whether the biomarker (for example elevated IL-6) is simply an indicator of disease or if it is actually causal or in the causal pathway of disease. In atherosclerosis, there is evidence to suggest that cytokines and the immune cells they attract may play an active role in disease. Cytokines and chemokines such as IL-1β and TNFα can activate vascular endothelial cells causing upregulation of adhesion molecules like Vascular Adhesion Molecule 1 (VCAM-1) (Libby, 2000). They can also attract leucocytes that can then enter the artery wall and start an atherosclerotic lesion or plaque. Macrophages in the plaque begin to store lipids and form “foam cells” that are characteristic of an early atheromatous precursor. Interferon-gamma (IFN-γ) produced by T cells or macrophages can induce vascular cells to produce the chemokines IFNγ-induced protein 10 (IP-10), CXC motif chemokine ligand 9 (CXCL-9), and IFN-inducible T cell alpha chemoattractant (I-TAC) further attracting inflammatory cells of the immune system. As plaques progress, they begin to accumulate calcium (Libby, 2000).

Atheromatous plaques can be stable for years, but when the plaques rupture, the resulting thrombosis can result in a cardiac event (Libby, 2000). Vulnerable plaques that are more susceptible to rupture usually have large numbers of activated T cells and macrophages within the plaques (Libby, 2000). In addition to IFN-γ, IL-6 may also be involved in plaque rupture (Libby, 2000; Kritchevsky et al., 2005). Studies are now showing the involvement of immune cells and cytokines in all stages of atherosclerosis, from plaque formation through thrombosis, plaque rupture and the cardiac event (Rea et al., 2018). The initiation of inflammation surrounding and contributing to CVD is not completely understood. Studies in mice have shown that even germ-free mice are susceptible to atherosclerosis suggesting that “sterile” inflammation may also play a part in inflammation related to atherosclerosis. In a mouse model of diet-induced atherosclerosis, deposits of cholesterol crystals were found in the atherosclerotic lesions (Duewell et al., 2010). The study demonstrated that cholesterol crystals could trigger NLRP3 inflammasome formation and attract inflammatory immune cells. The authors also saw cholesterol crystals in plaques from human patients. To investigate further the role of cholesterol and IL-1β, peripheral blood mononuclear cells (PBMCs) were primed with LPS then incubated with cholesterol crystals. This resulted in the release of cleaved mature IL-1β (Duewell et al., 2010). Importantly, these studies showed deposits of cholesterol crystals early in the process coinciding with the accumulation of immune cells (Duewell et al., 2010).

Lastly, in a large clinical study of 10,061 patients with a previous myocardial infarction, treatment with the anti-IL-1β monoclonal antibody, Canakinumab, resulted in reduced myocardial infarctions (Ridker et al., 2017). This was a placebo-controlled study and three doses of Canakinumab were tested, 50mg, 150mg, and 300 mg. The primary end-point of the study was nonfatal myocardial infarction, nonfatal stroke, or cardiovascular death. With increasing Canakinumab doses there were decreasing numbers of end-point cardio events. Interestingly, this effect was seen even with no reduction in lipids (Ridker et al., 2017). One noteworthy drawback was the association of Canakinumab treatment with a greater incidence of fatal infections (Ridker et al., 2017).

### Aging and Cancer

Both inflammaging and immuno-senescence that characterize aging are well-recognized carcinogens and are major risk factors for cancer development. More than 30% of all cancers are diagnosed among people above age 60 (Chatsirisupachai et al., 2019; White et al., 2019). The convergence of longer survival of aged individuals with advances in medical care in general, and higher predisposition to cancers in elderly may lead to further increases in cancer diagnosis among the elderly. The defects in anti-cancer immuno-surveillance mechanisms that accrue with age trigger the multi-stage process of cancer development in this population. Specifically, an imbalance between pro-inflammatory and anti-inflammatory mechanisms in elderly individuals contributes to increased susceptibility to cancer (Scaffidi and Misteli, 2006; Jose et al., 2017; Harari and Kupiec, 2018). Aged mice are also more susceptible to early dysplasia and oral cancer development in a 4-Nitroquinoline 1-oxide (4-NQO) model of oral carcinogenesis (Bhaskaran et al., 2021a). Because different subsets of CD4^+^ T cells and their functions determine the outcomes of mucosal barrier immunity, T-dependent B cell responses, and CD8^+^ T cell-mediated anti-tumor immunity, alterations in these cells contribute to hampering of anti-cancer responses in the elderly. Higher expression of immune inhibitory receptors (as discussed below) in CD4^+^ T cells is one of the characteristic features in aged mice (Channappanavar et al., 2009). Thus, on the one hand, attenuated CD4^+^ T cell function could provide an immune-tolerant microenvironment, allowing cancer cells to evade anti-tumor responses. However, no clear consensus has been reached in defining the precise role of the cells expressing inhibitory receptors in governing immuno-surveillance. Chronic inflammation coinciding with CD4^+^ T cell hyperactivation, on the other hand, is also associated with increased risk and mechanisms of initiation, progression, and metastatic diffusion of cancers. Activation of the nuclear factor kappaB (NF-kB) and STAT signaling are hallmarks of inflammatory responses that are frequently detected in tumors (Pikarsky et al., 2004; Karin, 2006; Bollrath and Greten, 2009). IL-6 and IL-1β, two of the cardinal senescent cytokines, play central roles in tumor initiation and growth (El-Omar et al., 2000; Becker et al., 2004; Huang et al., 2019; Hamarsheh and Zeiser, 2020), partially explaining the aging-associated susceptibility to cancers. In the colon carcinogenesis model induced by colonic inflammation, tumor cell-derived soluble IL-6 receptor is required for the progression of inflammation and cancer (Becker et al., 2004). IL-1β is up-regulated in breast, colon, lung, oral, and esophageal cancers (Escobar et al., 2015; Shiiba et al., 2015; Voronov and Apte, 2015; Garon et al., 2020), and linked to poor prognosis for patients with esophageal cancer (Chen et al., 2012). We have previously shown that increased IL-1β in the oral tumor milieu in aging mice correlated well with earlier progression of dysplasia and tumor development in tissues (Bhaskaran et al., 2021a)*.* Targeting IL-1β has been shown to interrupt oral carcinogenesis in the mouse model, and is being considered in the clinical setting (Dinarello, 2010; Wu et al., 2016). While tissue macrophages instigate initial inflammatory responses producing these cytokines, prolonged activation of pro-inflammatory T cells was required for sustained inflammation and tumor progression (Karin, 2006). In the context of aging, senescent T cells are usually capable of producing excessive amounts of inflammatory cytokines (Nakagami, 2020). Thus, it is tempting to speculate that senescent CD4^+^ T cells and cytokines produced by them, among other components, could play a critical role in promoting an environment more conducive for tumor development and growth.

In addition to inflammatory cytokines, immune-suppressive cells including tumor-associated macrophages (TAM), myeloid-derived suppressive cells (MDSC) (Bronte et al., 2016; Grzywa et al., 2020), and T_reg_ cells accumulate in tumors and are thought to contribute to poor immunologic responses against them. Heightened T_reg_ accrual and elevated T_reg_/CD8^+^ T cell ratios are commonly found in the tumor microenvironment (Mandal et al., 2016; Bhaskaran et al., 2021a), and can also hinder the success of *α*-PD1 cancer immunotherapy (Pandiyan and Lenardo, 2008; Barron et al., 2010; Wang et al., 2012; Pierson et al., 2013; Tai et al., 2013; Nowicki et al., 2018; Wen et al., 2019; Tay et al., 2020). We have previously shown that MDSC and T_reg_ accumulation were found in both mouse and human oral tumor tissues and were more pronounced in aged mice (Bhaskaran et al., 2021a)*.* These cells correlated with an increased IL-1β expression in the context of carcinogenesis. Although Foxp3^+^ ROR-γt^+^ cells were also shown to be present in tumors and contribute to tumor immune evasion and autoimmunity control (Downs-Canner et al., 2017; Kim et al., 2017; Bhaskaran et al., 2021a), the role of increased IL-1β in the context of aging and carcinogenesis remains to be investigated. MDSCs are of two types: monocytic and poly-morphonuclear, and produce T cell inhibitory factors such as arginase-1 enzyme. These cells have been shown to correlate with aging associated immuno senescence and tumor pre-disposition (reviewed elsewhere) (Fane and Weeraratna, 2020). Because aging is also associated with reduced microbiota diversity, changes in entero-bacteria, as well as an increase in certain types of bacteria and fungi, these could result in local immunological changes promoting tumor growth and progression (Zapata and Quagliarello, 2015; Feres et al., 2016; Buford, 2017; García-Peña et al., 2017; Shibagaki et al., 2017; Clark and Walker, 2018). The potential role of dysbiosis in dysregulating the above-mentioned cells requires detailed studies in geriatric conditions. These studies should lead the way to the potential preventive and therapeutic role of the modulation of the cancer-associated inflammatory microenvironment in the context of aging.

## Cellular Alterations in Aging

Hematopoietic changes with aging result in a shift from lymphoid to myeloid lineage differentiation in the bone marrow (Pang et al., 2011). Bone marrow from the elderly have higher frequencies of hematopoietic stem cells (HSC) compared to HSC from young bone marrow and HSC from the elderly have a myeloid-biased differentiation potential and are less quiescent (Pang et al., 2011; Denkinger et al., 2015).

Though there is an increase in the proportions of myeloid cells (macrophages, neutrophils, dendritic cells) with age, their functions are altered (Nikolich-Zugich, 2018; Oh et al., 2019). Phagocytic function of neutrophils and macrophages declines with age and dendritic cell uptake of antigen and expression of costimulatory markers declines with age as well (Nikolich-Zugich, 2018; Oh et al., 2019). Age-related dysfunction in innate immunity is reviewed in more detail elsewhere (Shaw et al., 2013).

One key alteration in immune cells with aging is a shift in the proportion of T cell maturation subsets. There is a decrease in the proportion of naïve T cells and a subsequent increase in proportions of memory T cells (Nikolich-Zugich, 2018; Mittelbrunn and Kroemer, 2021). There is also a decrease in the absolute number of naïve CD4 and CD8 T cells (Nikolich-Zugich, 2018; Thyagarajan et al., 2021). Decades of pathogen exposure in the elderly drives memory-T cell differentiation and thus the increased proportions of memory cells. CMV infections in particular result in an increase in CD8 memory T cells (Freeman et al., 2016b; Thyagarajan et al., 2021). Once infected, CMV remains with the host life-long and must be controlled by the immune system. CMV latent reservoirs reside mostly in salivary glands and lungs and may reactivate during times of immune compromise (Voigt et al., 2018). The likelihood that someone is positive for CMV increases as a person ages. An increase in CD8 memory cells, driven by CMV infection, likely influences the CD4/CD8 T cell ratio and the IRP in the elderly (Wikby et al., 2002).

Further, thymus atrophy is a critical contributor to changes in the cellularity of T cells. It is not clear when thymic atrophy begins (generally around puberty), but it is clear that by the age of 75 the human thymus is virtually non-existent and turns to fat tissue. As a result, remaining peripheral naïve T cells undergo homeostatic proliferation in an attempt to maintain their numbers (Goronzy and Weyand, 2017). Each time a cell divides there is a risk for DNA damage, with continuous replication, even homeostatic proliferation can result in a build-up of DNA damage, chromosome instability, and possibly aneuploidy (an unbalanced karyotype) (Barroso-Vilares and Logarinho, 2019). Aberrant DNA replication and the resulting accumulation of DNA damage is one of the main triggers of senescence (Di Micco et al., 2021). Several studies have also found a correlation between defective chromosome segregation and cellular senescence (Barroso-Vilares and Logarinho, 2019). When chromosome damage occurs, cytoplasmic chromatin fragments trigger the cytosolic DNA sensor cyclic GMP-AMP synthase. This leads to the activation of the Stimulator of Interferon Genes (STING) that activates inflammatory pathways (Barroso-Vilares and Logarinho, 2019).

Interestingly, the proinflammatory cytokines IL-6 and TNFα were shown to decrease thymopoiesis (Sempowski et al., 2000; Gruver and Sempowski, 2008; Mittelbrunn and Kroemer, 2021). IL-6 mRNA levels in the thymus were correlated with decreased levels of thymopoiesis in older and myasthenia gravis patients (Sempowski et al., 2000).

### Immunosenescence, in General, and T Cell Senescence, Specifically

Immunosenescence is a general term meaning the declining function of the immune system with age (Pawelec, 2018). A host of health concerns result from this phenomenon; declining surveillance for cancer, poor response to vaccination, heightened inflammation, disruption of immune homeostasis, slower recovery from injury, and autoimmunity, to name a few. Although immunosenescence is associated with aging and diseases such as chronic HIV (Fernandez et al., 2006; Shive et al., 2015; Shive et al., 2021), identifying the mechanisms and causal pathways has been challenging. It is probable that many of the underlying mechanisms and pathways causing immunosenescence are similar in aging and chronic viral infections such as HIV.

The classic definition of cellular senescence is “a state of dysregulated function associated with growth cycle arrest” (Feehan et al., 2021). Replicative cellular senescence was first described in cultured fibroblast by Hayflick and Moorhead in 1961 (Hayflick, 1961). A strict definition of cellular senescence would characterize the cell as no longer capable of proliferating, ever. However, there are often external factors that cause cells to appear senescent. When those factors are removed, for example when the cells are cultured *in vitro* away from the *in vivo* microenvironment, they may be released from growth inhibition.

Although senescent cells are regarded as unable to replicate in response to growth stimuli they are still metabolically active and survive. In fact, many senescent cells produce high amounts of immune mediators such as IL-6, IL-8, IL-1β, TGFβ, and GM-CSF. These molecules vary depending upon senescent cell type and are referred to as senescence-associated secretory phenotype (SASP) (Coppé et al., 2008). Senescent cells cannot be identified by a single biomarker, but instead are identified by a combination of markers. One marker included in the identification of non-immune senescent cells is senescence-associated-b-galactosidase (SA-b-gal). Nuclear senescence-associated heterochromatin foci (SAHF) and accumulation of p16 are also used to identify non-immune senescent cells; however, these markers are not exclusive. Non-immune cells such as neurons, adipocytes, osteocytes, and hepatocytes have the cellular machinery to undergo cellular senescence (Barroso-Vilares and Logarinho, 2019). An outstanding principle about senescent phenotypes is that they are very heterogeneous and the markers vary depending upon cell type and how senescence was triggered (Di Micco et al., 2021).

T cells in particular become senescent with age and lose their functional abilities to mount or regulate the immune response and maintain homeostasis, generate memory, and respond to new pathogens. Many attempts have been made to find the best flow cytometry markers to identify senescent T cells. Characteristics of T cell senescence are decreased telomerase activity and shortening of telomeres, low proliferative activity, and expression of senescence-associated markers CD57 and KLRG-1 (and intracellular molecules p38 and gH2AX) (Rodriguez et al., 2020). The accumulation of CD57^+^ and KLRG-1+ T cells increases with age (Bandrés et al., 2000; Ouyang et al., 2003; Focosi et al., 2010; Kared et al., 2016; Mittelbrunn and Kroemer, 2021) and is pronounced in HIV infection (Palmer et al., 2005; Wang et al., 2020) even after successful ART (Fernandez et al., 2011; Shive et al., 2021).

KLRG-1 is an NK cell marker, but when expressed on T cells it may indicate immune senescence. KLRG1 possesses an immune receptor tyrosine-based inhibitory motif (ITIM) in its cytoplasmic domain, suggesting inhibitory function ability when binding to its ligand E-cadherin (Henson and Akbar, 2009; Li et al., 2009). When KLRG-1 signaling was blocked during TCR activation by using antibodies against its ligand, E-cadherin, proliferation was enhanced (Henson and Akbar, 2009). Although E-cadherin is expressed on epithelial cells and Langerhans cells, a variety of antigen-presenting cells can express E-cadherin. This may help explain how KLRG-1 signaling could result in inhibition and immune cell senescence (Henson et al., 2009).

CD57 is a glycoprotein used to identify senescent T cells. CD57 can bind L- and P-selectins but its mechanism of action remains unknown (Focosi et al., 2010). The cell surface expression of CD57 is elevated on T cells from the elderly (Bandrés et al., 2000; Focosi et al., 2010; Mittelbrunn and Kroemer, 2021) and HIV-infected patients and is associated with decreased proliferative ability (Brenchley et al., 2003). One study that examined the function of CD57 ^+^ T cells in HIV disease found that CD8 T cells that did not proliferate after antigen stimulation expressed CD57 (Brenchley et al., 2003). They confirmed this lack of proliferation in CD57 ^+^ T cells by stimulating CD8 T cells from HIV uninfected as well as HIV-infected participants with the super-antigen SEB. This inability to proliferate in response to antigen persisted in the CD57 ^+^ T cells even after addition of IL-15 (Brenchley et al., 2003). To further characterize these cells as senescent, they examined telomere length and found that the CD57 ^+^ CD8 T cells had shortened telomeres (Brenchley et al., 2003). However, recent data contradict these earlier findings. Studies that examined CD4 memory T cells from uninfected, HIV negative donors, found that CD57 ^+^ T cells could proliferate in response to IL-15 stimulation (Focosi et al., 2010; Ahmed et al., 2020; Chen et al., 2020). However, these studies did not examine antigen-specific CD8 T cells.

In a young, healthy immune system, naïve T cells express CD45RA and CCR7 that allows them to home back to lymph nodes where they “search” for antigen-presenting cells (APCs) expressing “their” cognate antigen. In the lymph node, if naïve T cells encounter their cognate antigen, they receive proliferative signals; if not, they receive survival signals and continue to circulate through the lymphatic system. Interleukin-7 is a homeostatic cytokine that is specifically necessary for naïve T cell survival and proliferation. IL-7 signals through a heterodimeric receptor composed of the common gamma chain (CD132) and the IL-7 receptor alpha chain, CD127. If the naïve T cell encounters cognate antigen via its T cell receptor (TCR) presented by a professional APC it will receive additional costimulatory signals through CD28 and CD27 on its surface.

T cell expression of CD127 is lower in the elderly (Kim et al., 2006) and plasma IL-7 is elevated (Marttila et al., 2011), possibly because the IL-7 is not being bound by CD127 + T cells. As mentioned above, the proportion and number of naïve T cells in elderly are reduced (Nikolich-Zugich, 2018; Mittelbrunn and Kroemer, 2021; Thyagarajan et al., 2021). Like inflammaging in the elderly, treated HIV infection is characterized by chronic elevated systemic inflammation (Freeman et al., 2016a; Sieg et al., 2021) that is even higher in those patients that have difficulty recovering their CD4 T cell counts (Shive et al., 2015). Elevated plasma levels of IL-6 is at the forefront of both aging and chronic viral infection. *In vitro* studies examining the effects of IL-6 and IL-1β on the IL-7 axis found that incubation of healthy PBMCs with IL-6 or IL-1β resulted in a decrease of CD127 on the cell surface and transcriptionally and this effect was most pronounced in naïve T cells (Shive et al., 2014). In addition, if cells were pretreated in culture with IL-6 or IL-1β and then IL-7 was added, upregulation of the survival factor Bcl2 was attenuated (Shive et al., 2014). It is possible that a similar mechanism may be at play in the elderly and elevated IL-6 may contribute to lower T cell expression of CD127. This, in turn, may be contributing to decreased naïve T cell numbers and greater naïve T cell apoptosis in the elderly.

After naïve T cells encounter their antigen and clonally expand, they become effector cells and lose the expression of CCR7, CD27, and CD28. The loss of CD27 and CD28 is accompanied by an increase in p53-induced p21. P21 has been associated with telomere damage, and p16 was associated with cellular stress and premature senescence (Xu and Larbi, 2017). P21 and p16 regulate the cell cycle by inhibiting cyclin-dependent kinases and preventing the progression from G1 to S during cell cycle (Adams, 2009; Xu and Larbi, 2017). This could explain the beginning cascade of T cell senescence. If stimulation continues, effector T cells may undergo apoptosis or become terminal effector (TE) memory T cells. Terminal effector memory T cells reacquire the expression of CD45RA but remain CCR7, CD27, and CD28 negative (Xu and Larbi, 2017). TE memory T cells may have a lower or no proliferative capacity, but continue to secrete cytokines and begin to express “senescence markers” such as CD57 and KLRG-1 and the molecular marker T-bet (Dolfi et al., 2013; Xu and Larbi, 2017). T cells in a replicate senescent state are most often phenotypically effector memory (EM) and TE memory T cells. *In vitro* stimulation of PBMCs with inflammatory cytokines IL-6 and IL-1β can induce the surface expression of CD57 and PD-1 on CD4^+^ T cells after 7 days. However, IL-7 can also induce PD-1 and CD57 expression (Shive et al., 2015). Further studies are needed to determine if these cells are truly exhausted or senescent and if such dysregulation is seen in the elderly.

### T Regulatory Cell Dysfunction in the Elderly

T_regs_ are central to immune tolerance against autoimmunity and commensal microbes, immunomodulation during infection and inflammation, and tissue homeostasis (Pandiyan et al., 2011a; Josefowicz et al., 2012; Pandiyan and Zhu, 2015; Pandiyan et al., 2016; Pandiyan et al., 2019; Bhaskaran et al., 2021b). Therefore, the proportion and function of these cells have been investigated in the context of inflammaging in many studies. Although the production of thymically derived T_regs_ declines with age (Goronzy and Weyand, 2017), the periphery shows heightened prevalence of T_regs_ despite an exaggerated inflammatory state in the elderly (Tsaknaridis et al., 2003; Lages et al., 2008; Huynh et al., 2015; Raynor et al., 2015; Pulko et al., 2016; Del Giudice et al., 2018; Nikolich-Zugich, 2018). Increased accumulation of T_regs_ with low levels of CD25 and high levels of PD-1, inducible T cell co-stimulator (ICOS), and CTLA-4 is shown to be associated with increasing levels of pro-inflammatory IL-6 as well as declining levels of IL-2 that are observed during aging (Channappanavar et al., 2009; Raynor et al., 2013; Raynor et al., 2015; Li and Xiong, 2020). IL-6 appears to upregulate ICOS that plays a role in aged T_reg_ differentiation, survival, and proliferation (Nishioka et al., 2006; Raynor et al., 2015; Li and Xiong, 2020). Association of heightened levels of T_regs_ with aging-associated inflammation initially suggested that T_regs_ from aged mice may not be suppressive. However, aged T_regs_ from lymph nodes were found to be suppressive, and in fact, exhibit augmented suppressive activity per cell as compared to young T_regs_ in mice (Lages et al., 2008). Other studies examined their function in aging-associated immuno-senescence. By measuring lymph node T_regs_, it was found that T cell-mediated immuno-senescence is ascribable to changes in the non-T_reg_ T cells but not to a functional and proportional enhancement of suppressive T_regs_ (Nishioka et al., 2006; Sefik et al., 2015; Harpaz et al., 2017; Bhaskaran et al., 2020). In aged mucosa, however, T_regs_ are found to be dysfunctional (T_regDys_) (Bhaskaran et al., 2020). They expand in response to IL-6, upregulate the effector cytokine IFN-γ, and may lack immunomodulatory functions. The increase in this population of FOXP3^+^ cells directly correlates with increased inflammation in the context of infection response (Bhaskaran et al., 2020). Recent studies show phenotype and functional heterogeneity as well as plasticity in T_regs_ (Pandiyan and Zhu, 2015). Many FOXP3^+^ subsets are now known to express non-canonical transcriptional factors and cytokines associated with inflammation and have distinct functions in mucosa and tissues (Kitani and Xu, 2008; Lochner et al., 2008; Sefik et al., 2015; Kitz et al., 2016; Di Giovangiulio et al., 2019). FOXP3+ cells expressing T-bet and ROR-γt have also been shown to have little or no suppressive activity in tissue niches in infection and in tumors (Santegoets et al., 2019; Bhaskaran et al., 2020; Bhaskaran et al., 2021a). Such cells are dysfunctional during inflammation, and their functional heterogeneity is strongly regulated by the local cytokine milieu, the details of which remain unclear. However, in other instances, FOXP3+ cells exploit these transcription factors to differentiate into specialized T_reg_ cell populations within the periphery and restrain their target cells expressing the same transcription factors and the corresponding type of immune response. For example, transcription factor T-bet controls regulatory T cell homeostasis and is required for its suppressive function during type I inflammation (Koch et al., 2009). Similarly, IRF-4 and GATA-3, Th2 transcription factors, are necessary for T_reg_ cell-mediated control of Th2 cell responses (Zheng et al., 2009; Wohlfert et al., 2011). Also, FOXP3+ T_regs_ express and require STAT3, a transcription factor driving T helper 17 (Th17) cell differentiation, in order to suppress Th17 cells (Chaudhry et al., 2009; Pandiyan et al., 2011a; Pandiyan and Zhu, 2015). RORγt-driven CD161 + T_reg_ cells are a specialized population that controls inflammation and epithelial barriers, supporting wound repair in intestinal mucosa (Povoleri et al., 2018). We have previously shown that RORγt^+^ T_regs_ (T_reg_17) harbor mucosal immunomodulatory functions and are maintained by the MyD88-IL-1β axis (Bhaskaran et al., 2020). With aging and local impairment of IL-1β, this axis becomes dysregulated causing a specific loss of these mucosal T_reg_17, despite an increased accumulation of FOXP3+ cells. Thus, the T_reg_/T_reg_17 mediated immune-regulation arm is impaired during aging (Bhaskaran et al., 2020). Accumulated FOXP3+ cells are of T_regDys_ phenotype and expand with IL-6 in the milieu, which is consistent with the previously demonstrated effect of IL-6 in expanding IFN-γ^+^ cells (Strutt et al., 2016). Loss of T_reg_17 and increase in T_regDys_ in mucosa significantly correlates with an exaggerated inflammation and hyperproduction of IFN-γ by T cells during infection. A recent study showed that T cell IFN-γ hyper-production contributes to exaggerated immunopathology and may facilitate susceptibility to mucosal fungal infection by impairing the integrity of the epithelial barrier (Break et al., 2021; Kisand et al., 2021). Although this may not be primarily responsible for the *Candida* susceptibility in the mucosa (Kisand et al., 2021; Puel and Casanova, 2021), excessive IFN-γ expression caused by T_reg_ dysregulation might secondarily affect the mucosal barrier competence in aged mucosa. This may be due to the detrimental effect of IFN-γ on epithelial cells and barrier integrity (Beaurepaire et al., 2009; Dolowschiak et al., 2016), although the role of this mechanism needs to be investigated in immuno-senescence and aging. Thus, age-dependent immune dysregulation could be attributed to the dysfunction of specific-FOXP3+ subsets due to an imbalance in cytokines that regulate them in local tissue niches.

CD25^low^ FOXP3^+^ cells found in aged mucosa may represent a subset of exhausted T_regs_ expressing PD-1 and Ki-67 and expanding in response to an inflammatory reaction in tissues (Ferreira et al., 2017). One of the mechanisms by which T_regs_ function involves their ability to consume IL-2, which leads to conventional CD4^+^ T cell apoptosis, down-regulation of IL-2 receptor (CD25) and IFN-γ production, and up-regulation of IL-17A depending on the cytokine milieu (Pandiyan et al., 2007; Pandiyan et al., 2011b; Chinen et al., 2016; Bhaskaran et al., 2020). In addition to T_reg_17 cell loss during aging (Bhaskaran et al., 2020), excessive IL-6 may additionally dysregulate suppression by T_regs_ because IL-6 is known to act on conventional CD4^+^ T cells and render them less sensitive to the IL-2 consumption-mediated suppressive effect of T_regs_ (Pandiyan et al., 2007; Pandiyan et al., 2011a; Nish et al., 2014). Furthermore, reduced CD25 expression in aged FOXP3^+^ cells and their impaired ability to compete for IL-2 may also contribute to T_reg_ dysfunction and immunopathology during infection in the elderly (Scheffold et al., 2005; Pandiyan et al., 2007; Wang et al., 2010; Chinen et al., 2016). Taken together, the above-described studies support a paradigm that cytokine imbalance occurring during acute infection or dysbiosis in mucosa may contribute to T_reg_ dysregulation worsening the overt inflammation in elderly individuals. They also suggest that manipulating the underlying signaling components regulating these pathways may offer potential strategies to combat immune dysfunction in human aging.

## Concluding Remarks

**FIGURE 1:**
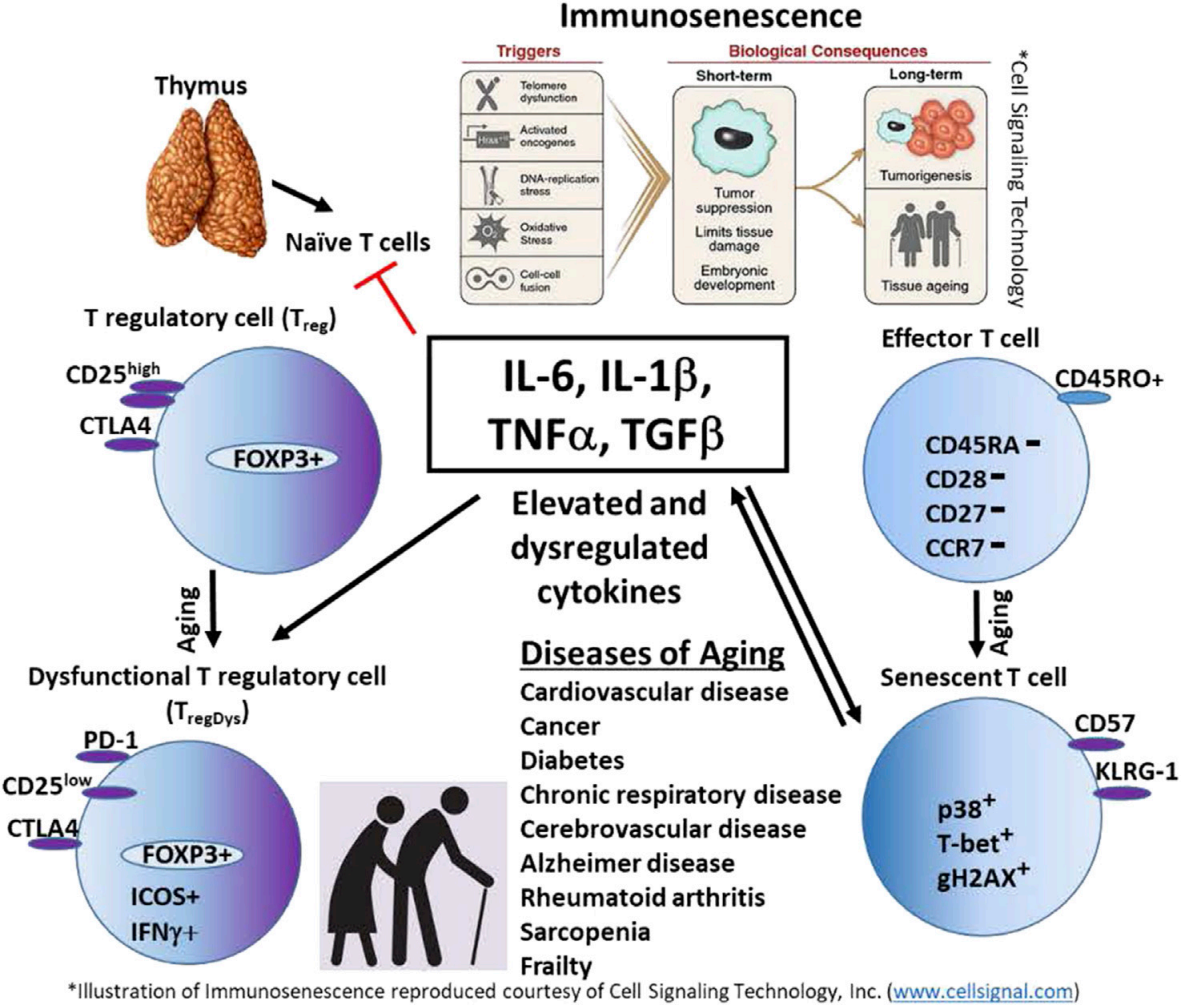
Model. Persistent elevation of innate cytokines such as IL-6, IL-1β, TNFα, and TGF-β, contribute to immunosenescence in the elderly. These cytokines also promote T cell senescence. Senescent T cells perpetuate inflammation by secreting inflammatory cytokines. IL-6 also may promote the development of dysfunctional regulatory T cells (T_regDys_), indirectly contributing to chronic inflammation in the elderly. The result is aging and immune and non-immune diseases of aging.

The human immune system is designed to recognize and clear pathogens, damaged or abnormal cells and debris and generate a memory response to more effectively clear the pathogen on the next encounter. During an immune response, a significant amount of “collateral damage” results. Just as importantly, the immune system is also designed to stop the inflammatory response and return the immune system to homeostasis. Much of the coordinated immune response and recovery is orchestrated by cytokines (Figure 1. Model). With aging, the delicate balance of immune activation and regulation is disrupted. It is clear that there is persistent low-level inflammation during aging, paradoxically, there also appears to be increased anti-inflammatory cytokines and elevated peripheral T_reg_ cells. Continuous exposure to immune stressors results in senescent cells that further contribute to ongoing inflammation by secretion of SASP. An alternate explanation for the persistently elevated inflammatory and anti-inflammatory cytokines is the development of T_regDys_ cells with aging. Understanding the mechanisms that drive immune senescence in aging will help guide the development of more effective treatments and prevent or postpone many of the diseases associated with aging. This will help close the gap between lifespan and healthspan and lead to healthy aging.
